# PSA-HWT: handwritten font generation based on pyramid squeeze attention

**DOI:** 10.7717/peerj-cs.2261

**Published:** 2024-08-23

**Authors:** Hong Zhao, Jinhai Huang, Wengai Li, Zhaobin Chang, Weijie Wang

**Affiliations:** 1 School of Computer and Communication, Lanzhou University of Technology, Lanzhou, Gansu, China; 2 School of Information Science and Engineering, Lanzhou University, Lanzhou, Gansu, China

**Keywords:** Handwriting font generation, Generative adversarial networks (GANs), Pyramid squeeze attention, Multi-scale feature, Long distance channel dependence

## Abstract

The generator, which combines convolutional neural network (CNN) and Transformer as its core modules, serves as the primary model for the handwriting font generation network and demonstrates effective performance. However, there are still problems with insufficient feature extraction in the overall structure of the font, the thickness of strokes, and the curvature of strokes, resulting in subpar detail in the generated fonts. To solve the problems, we propose a method for constructing a handwritten font generation model based on Pyramid Squeeze Attention, called PSA-HWT. The PSA-HWT model is divided into two parts: an encoder and a decoder. In the encoder, a multi-branch structure is used to extract spatial information at different scales from the input feature map, achieving multi-scale feature extraction. This helps better capture the semantic information and global structure of the font, aiding the generation model in understanding fine-grained features such as the shape, thickness, and curvature of the font. In the decoder, it uses a self-attention mechanism to capture dependencies across various positions in the input sequence. This helps to better understand the relationship between the generated strokes or characters and the handwritten font being generated, ensuring the overall coherence of the generated handwritten text. The experimental results on the IAM dataset demonstrate that PSA-HWT achieves a 16.35% decrease in Fréchet inception distance (FID) score and a 13.09% decrease in Geometry Score (GS) compared to the current advanced methods. This indicates that PSA-HWT generates handwritten fonts of higher quality, making it more practically valuable.

## Introduction

The task of handwritten font generation aims to mimic the handwriting style to produce realistic handwritten fonts and is an important application area of generative neural networks. Handwriting has widespread applications in daily life. Compared to rigid printed fonts, handwriting better reflects the personal characteristics of the writer. It is widely used in font style transfer, author recognition, handwriting signature verification, and other scenarios. The traditional font design process typically relies on professional designers to complete manually, which is time-consuming, labor-intensive, and costly. With the advancement of deep learning technology, automated handwritten font generation methods have gradually become a research hotspot. However, generating realistic handwritten fonts is a challenging task. Issues such as how to generate realistic handwritten fonts and how to achieve good generalization to new styles and new characters have not yet been fully resolved.

Currently, in the field of deep learning, methods for handwritten font generation can be broadly categorized into image-based offline methods and stroke-based online methods. Graves proposed an online font generation method based on LSTM neural networks (Graves, 2013). This method can predict the next stroke point based on input text and pen position information. Kotani, Tellex & Tompkin (2020) proposed an online stroke generation method based on RNN. This method encodes authorship, characters, and specific style variations in the RNN model to represent the style information of the font. Apart from the methods mentioned above, there are also some handwritten font generation methods based on generative adversarial networks (Goodfellow et al., 2020) (GANs). Haines, Mac Aodha & Brostow (2016) introduced a method that can infer handwritten characters with different styles from source images, but it’s restricted to generating characters within the source images. Alonso, Moysset & Messina (2019)’s research introduced a generative model that takes the input content string into account. This breakthrough overcomes the constraints posed by a fixed predefined vocabulary, leading to enhanced outcomes. However, this method often suffers from the problem of style collapse during training. Fogel et al. (2020) proposed the ScrabbleGAN, which employs a novel method to produce image widths that are directly related to the length of the input text and achieves good results on font content, but the generated fonts are not realistic enough. Davis et al. (2020) introduced an architecture based on StyleGAN, which works well by learning handwritten fonts generated based on styles and input text, but there is still room for improvement. GANwriting achieved the task of generating handwritten fonts with limited data sets by setting text content and style features under small samples (Kang et al., 2020). Kong et al. (2022) improved the quality of generated fonts by employing a component-based learning strategy to enhance local style representation learning. Pippi et al. (2023) proposed a method VATr, font generation is achieved by utilizing supervised pre-training on the dataset and representing the textual content as a sequence of visual archetypes (We have uploaded the literature on related work in tabular form to the Supplemental Materials).

**Figure 1:**
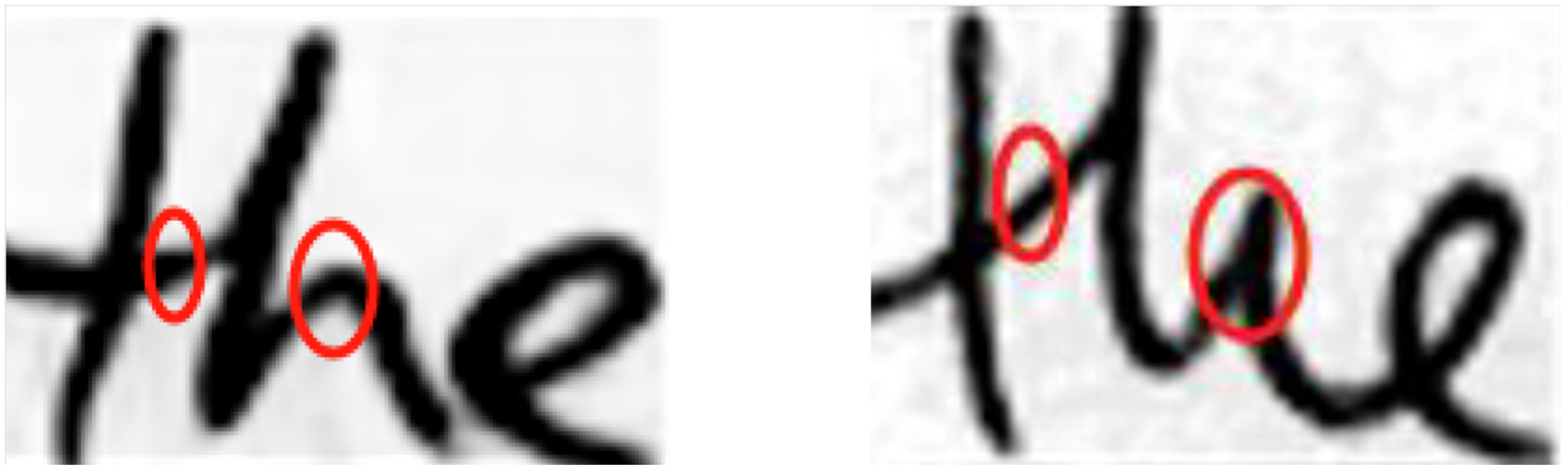
Comparison of generated fonts and real fonts. Images © IAM Handwriting Database, non-commercial use only.

The aforementioned methods for handwritten font generation often inadequately consider the importance of combining local and global features of handwritten fonts during feature extraction, resulting in poor stylistic performance in the generated handwritten fonts. For example, features such as the appearance of the font and the spatial relationships between the overall structure and strokes are not fully considered, leading to significant differences in content and style between the generated fonts and real ones. Bhunia et al. (2021) first proposed the Handwriting Transformer (HWT) model, which still utilizes a GAN network framework, with a combination of CNN (Albawi, Mohammed & Al-Zawi, 2017) and Transformer (Vaswani et al., 2017) as the generator to generate images. HWT achieves the fusion between style and content by obtaining style features for each query character. It captures the interactions between different letters in the style examples and the relationships between adjacent characters of the same letter through self-attention mechanisms. Additionally, HWT can handle arbitrary-length text and specified handwriting styles in a few-shot setting. It achieves this by employing a cross-attention mechanism between style representations and content tokens, resulting in better handwritten fonts compared to many previous GAN-based methods. However, the HWT encoder still struggles to effectively extract features such as stroke thickness and curvature at different scales when extracting sample features, resulting in significant discrepancies between the generated fonts and real handwritten fonts. The left image in Fig. 1 shows the font generated by the HWT model, while the right image displays real handwritten text. Comparing the left and right images in Fig. 1, it is evident that the font generated by the HWT model exhibits significant differences, particularly in the circled areas where the letters ‘the’ are located, indicating that the generated font has not effectively learned the stylistic nuances of real handwritten text.

Embedding attention modules in deep convolutional neural networks can effectively improve their performance. However, in handwritten font generation tasks, general attention mechanisms tend to focus only on the key information of the font contours, neglecting fine-grained features such as stroke thickness and curvature, which are present at multiple scales. In contrast, pyramid squeeze attention can not only integrate multi-scale information on each channel feature map using a multi-scale pyramid convolution structure but also establish long-term channel dependencies. Therefore, we employ pyramid squeeze attention to achieve multi-scale feature extraction. We employed extensive quantitative and qualitative experiments to evaluate our model, achieving excellent results and demonstrating its strong generalization performance.

## Materials and Methods

### Approach overview

To address the issue of the HWT encoder’s inability to effectively extract stroke details when extracting handwritten font features, inspired by the approach of embedding attention modules into convolutional neural network (CNN) networks to improve performance, we introduce Pyramid Squeeze Attention (PSA) (Zhang et al., 2022) into HWT, proposing a handwritten font generation model called PSA-HWT. We utilize ResNet50 (He et al., 2016) as the backbone network for the encoder. It incorporates Pyramid Squeeze Attention (PSA) into the ResNet50 network for multi-scale feature extraction. The features are then encoded using a Transformer encoder, and subsequently decoded by a Transformer decoder followed by a CNN decoder to generate handwritten fonts. The encoder of PSA-HWT performs multi-scale feature extraction on handwritten fonts using the PSA module. It captures spatial information from multi-scale input feature maps and establishes long-term dependencies between channels with multi-scale channel attention, thereby more fully extracting stroke details of the font. The Transformer encoder utilizes self-attention mechanisms to model features at different positions in the font sequence, capturing the relationships between each position and other positions’ features and enhancing its understanding of contextual information in the sequence, thereby improving the realism of the generated fonts. Additionally, a discriminator is used to distinguish between generated images and real images at both the pixel and global levels, encouraging the generator to produce more realistic images.

**Figure 2:**
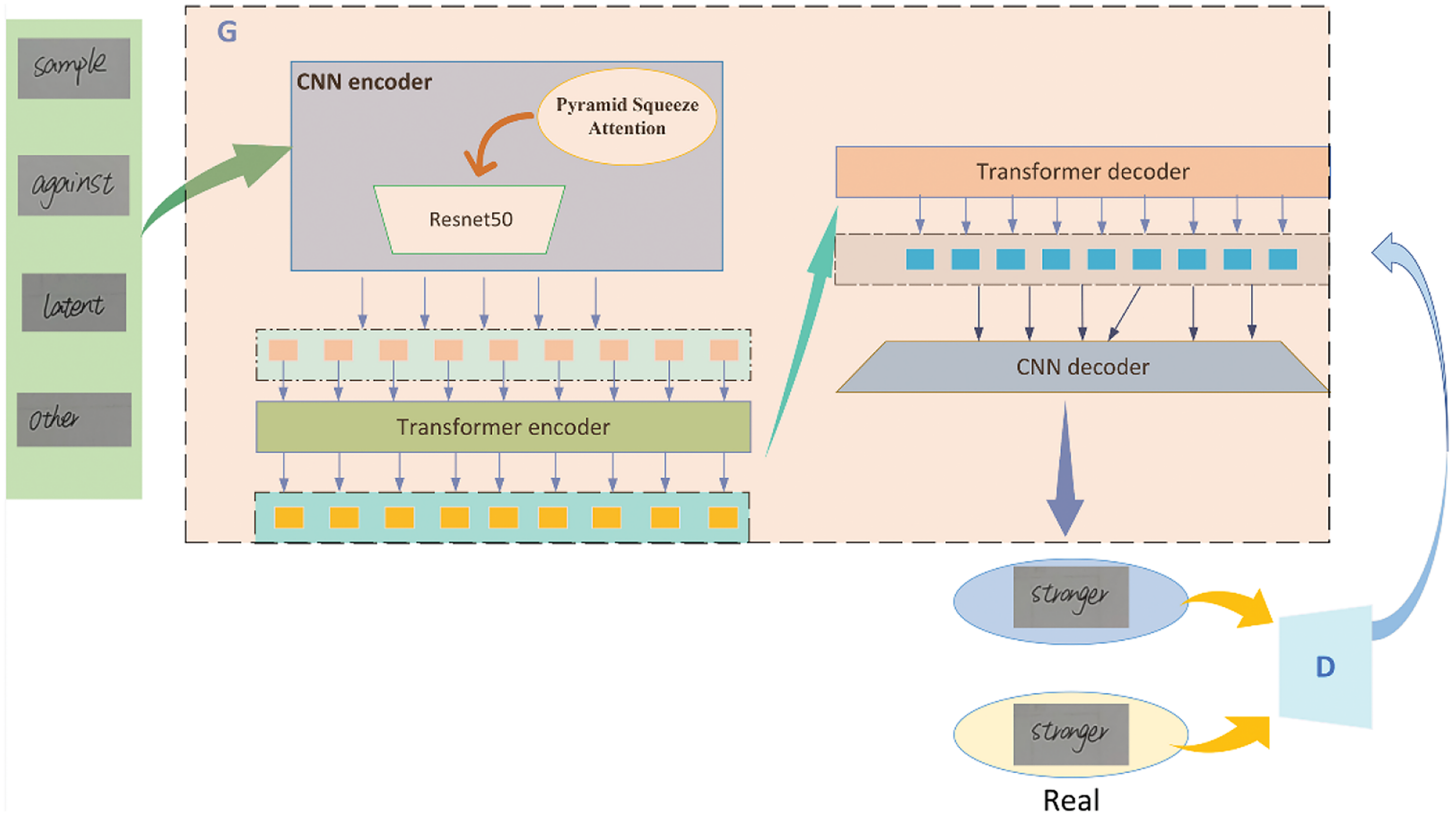
Overall architecture of our PSA-HWT. Images © IAM Handwriting Database, non-commercial use only.

PSA-HWT is mainly composed of G (Generator) and D (Discriminator), as shown in Fig. 2.

**Figure 3:**
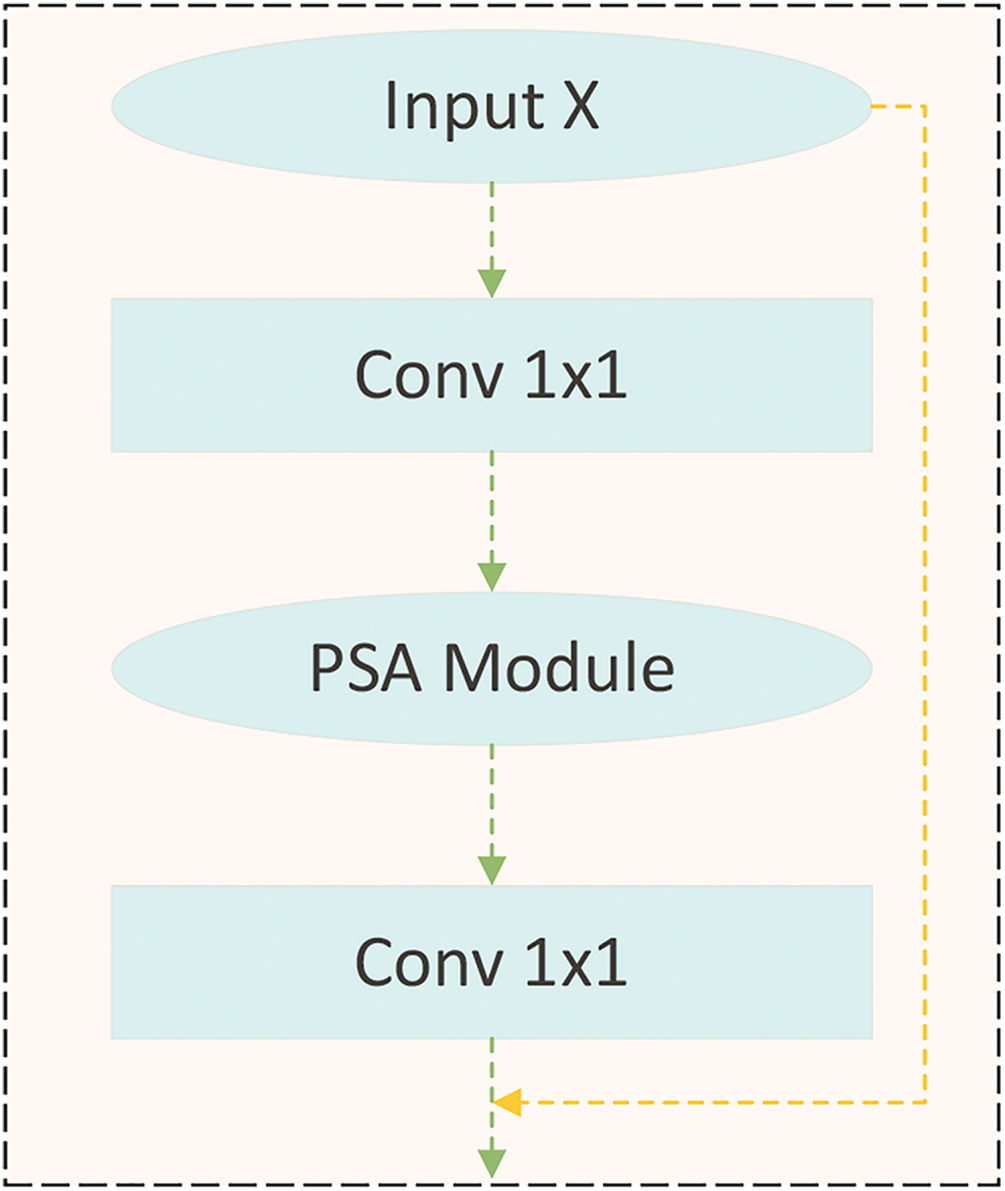
CNN encoder. Images © IAM Handwriting Database, non-commercial use only.

As illustrated by Fig. 2, generator G consists of two parts: an encoder and a decoder. The encoder is composed of a ResNet50 fused with pyramid squeeze attention and a Transformer encoder. The decoder consists of a Transformer decoder and a CNN decoder. The structure of the CNN encoder is shown in Fig. 3. The CNN encoder consists of the PSA module and ResNet50. In this configuration, one 3 $\times$ 3 convolution block in ResNet50 is replaced with the PSA module. The PSA module not only processes multi-scale input tensors using a multi-scale pyramid convolution structure but also compresses the channel dimension of the input tensors. This allows it to effectively extract spatial information of different scales from each channel feature map and place greater emphasis on the weights of the key information in the feature maps. This enables the model to fully extract the important features of the font strokes. First, we use a ResNet50 network incorporating pyramid squeeze attention generate lower-resolution feature maps ${h_{ij}} \in {{\mathbb{R}}^{h \times w \times d}}$ for each style image ${x_{ij}}$ (where $i$ represents a certain style, $j$ is the handwritten font image of the style, the variables $h$ and $w$ denote the height and width of the image, respectively, $d$ is the embedded size). Then, flatten the spatial dimensions ${h_{ij}}$ to produce the feature map sequence of size $n \times d$, where $n = h \times w$. After that, the feature sequence vectors derived from every style image are combined together to create a unified tensor ${H_i} \in {{\mathbb R}^{N \times d}}$, where $N = n \times P$. $P$ represents the set of the number of handwritten font images. The next step includes entering ${H_i}$ into the Transformer encoder to model the local and global combinations of feature sequences. The framework of Transformer encoder consists of several layers, encompassing both a module for multi-head self-attention and a module for multi-layer perceptron. The multi-head attention module within each layer is tasked with converting the input sequence from the preceding layer into a triplet, the triplet can be expressed as key $K$, query $Q$ and value $V$. The relationship between these three components is depicted in Formula (1).

(1) $$Q = {H^{l - 1}}{W^Q},K = {H^{l - 1}}{W^K},V = {H^{l - 1}}{W^V}$$

where ${W^Q} \in {{\mathbb R}^{N \times {d_q}}},{W^K} \in {{\mathbb R}^{N \times {d_k}}},{W^V} \in {{\mathbb R}^{N \times {d_v}}}$ are the learnable weight matrix for $Q$, $K$ and $V$ respectively. The calculation procedure for each attention head is shown in Formula (2).

(2) $${O^j} = softmax \left(\displaystyle{{Q{K^T}} \over {\sqrt {{d_k}} }}\right)V \in {{\mathbb R}^{N \times {d_v}}},j \in \left\{ {1, \cdots ,J} \right\}$$

Connecting the outputs $O = [{O^1}, \cdots ,{O^J}]$ of all $J$ heads through a $MLP$ layer yields the output feature sequence ${H^l}$ for the layer $l$. The final feature sequence $Z \in {{\mathbb R}^{N \times d}}$ is obtained after passing through a total of $L$ layers transformer encoder. Here, ${d_k}$ represents the dimension of $k$. Dividing by the square root of ${d_k}$ during the computation helps maintain gradient stability during training. We used the softmax function to normalize the results. The decoder consists of multiple Transformer decoder layers. The feature sequence $Z$ undergoes processing by multiple consecutive decoders to obtain feature vectors. In each decoder, embedded queries are processed in parallel. Finally, the feature vectors are concatenated and passed through a linear layer to obtain a vector matrix. This matrix is subsequently input into a CNN decoder comprising of four residual blocks and a Tanh layer, resulting in the production of the ultimate image of the handwritten font. Additionally, we replaced a 7 $\times$ 7 convolutional kernel in the ResNet50 network with three 3 $\times$ 3 convolutional kernels, which increases the network depth while reducing the number of parameters.

### Pyramid Squeeze Attention

**Figure 4:**
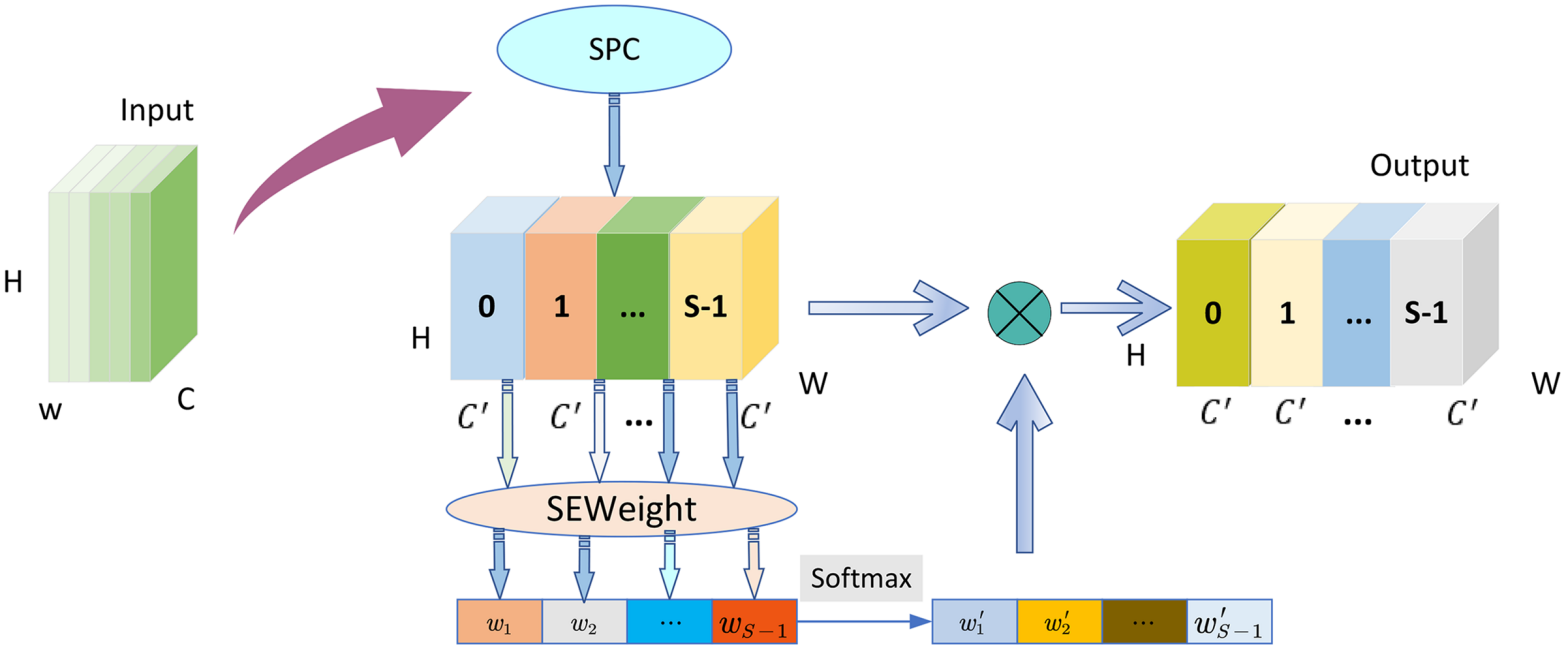
Pyramid squeeze attention. Images © IAM Handwriting Database, non-commercial use only.

Pyramid Squeeze Attention (PSA) efficiently captures and utilizes spatial information from feature maps at different scales. By employing a multi-scale pyramid convolution structure to extract features, it establishes longer-range channel dependencies, enabling the extraction of multi-scale features of the font at a finer granularity. This allows for better extraction of key features such as strokes, curvature, and thickness of the font. PSA is mainly composed of SEWeight (Hu, Shen & Sun, 2018) module and Squeeze and Concat (SPC) module, as shown in Fig. 4.

The SPC module can acquire multi-scale feature maps $H \times W \times {C^{\rm '}}$, where $H$ and $W$ represent the height and width of the input feature map respectively, and ${C^{\rm '}}$ are input channel dimensions. The channel attention on the feature maps at various scales is extracted utilizing SEWeight module, ${w_1},{w_2} \cdots ,{w_{S - 1}}$ is represented as channel attention vector, which is adjusted using Softmax to obtain the rescaled attention weight vector $w_1^\prime ,w_2^\prime \cdots ,w_{S - 1}^\prime$. Ultimately, the feature map is achieved through element-wise multiplication of the recalibrated weights and the corresponding feature map, generating a feature map with diverse scale information.

**Figure 5:**
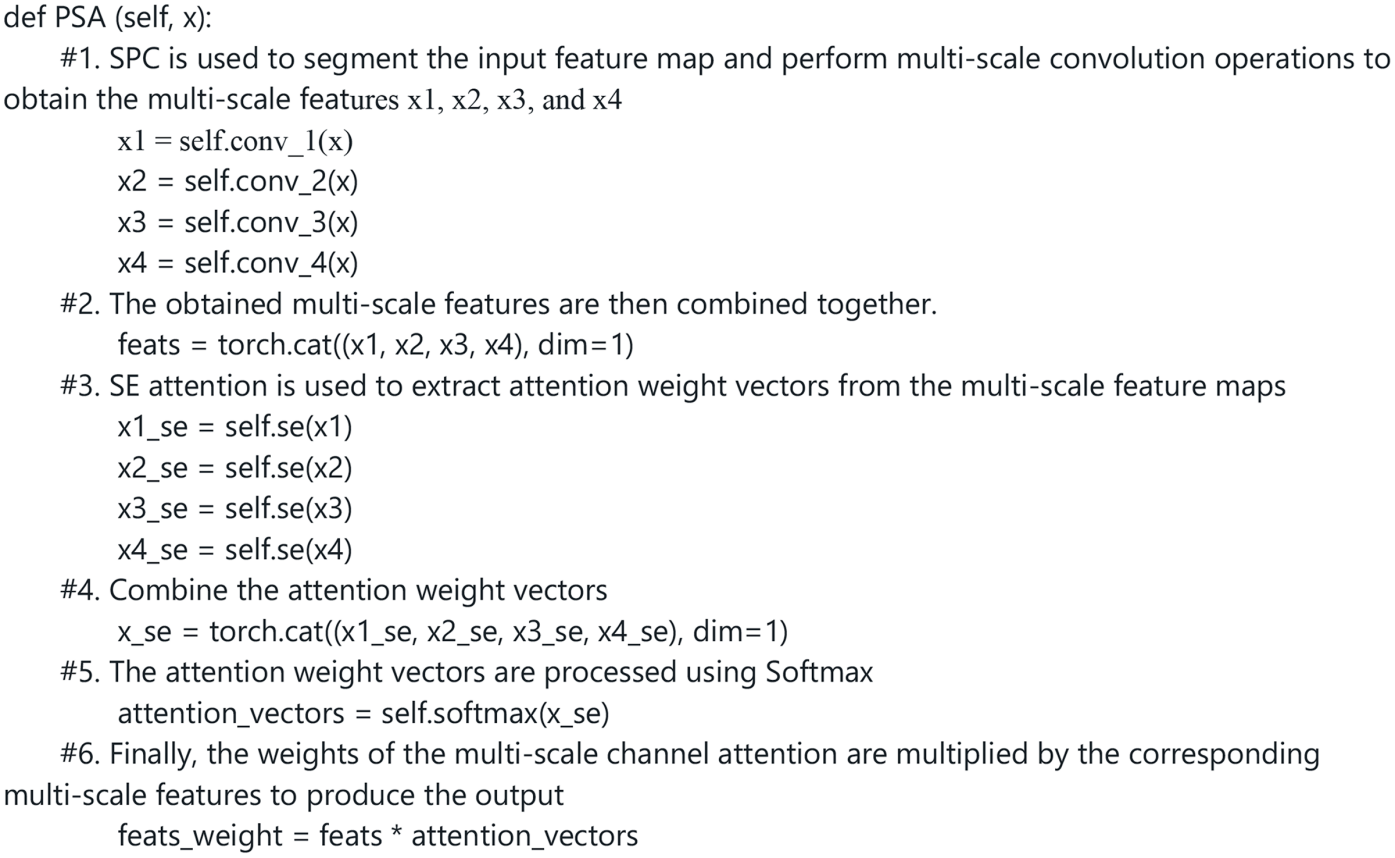
Pytorch code of the PSA module. Images © IAM Handwriting Database, non-commercial use only.

To help readers better understand the details of how pyramid squeeze attention achieves multi-scale feature extraction, Fig. 5 provides the main algorithm code and a detailed explanation.

### SEWeight module

**Figure 6:**
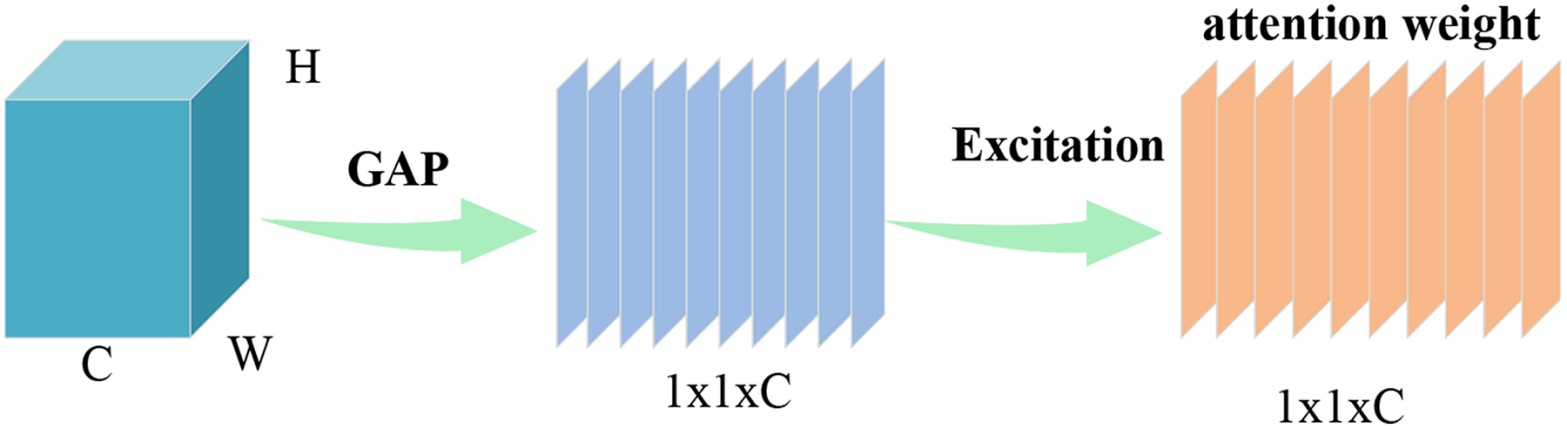
SEWeight module. Images © IAM Handwriting Database, non-commercial use only.

The channel attention mechanism can regulate the focus and weight allocation between different channels in the feature map, enhancing the model’s attention to specific channels. This improves the model’s ability to extract and represent important features. Let $X \in {{\mathbb R}^{C \times H \times W}}$ represent the input of the feature map, where $H$, $W$, and $C$ denote height, width, and the channel dimensions of the input, respectively. The SE block consists of two parts: squeeze and excitation, which are used to encode global information and adaptively recalibrate channel relationships, respectively. The SEWeight structure is shown in Fig. 6. Global average pooling is utilized to incorporate global spatial information into channel descriptors. Computation of the global average pooling operation is defined as,

(3) $${g_c} = \displaystyle{1 \over {H \times W}}\sum\limits_{i = 1}^H {\sum\limits_{j = 1}^W {{x_c}} } (i,j)$$

Equation (4) demonstrates the calculation of the attention weight for the $c$- $th$ channel in the SE block.

(4) $${w_c} = \sigma ({W_1}\delta ({W_0}({g_c})))$$

where $\delta$ represents ReLU and $\sigma$ represents Sigmoid. ${W_0} \in {{\mathbb R}^{C \times \textstyle{C \over r}}}$, ${W_1} \in {{\mathbb R}^{\textstyle{C \over r} \times C}}$. ${W_0}$ and ${W_1}$ are both fully connected layers, and the linear relationship between the channels is bound through ${W_0}$ and ${W_1}$.

We use the SEWeight module to allow the network to selectively weight the importance of each channel, accurately calculating the importance of each stroke position. This enables the model to better focus on the key features of the fonts, thus improving the quality of the generated fonts.

### SPC module

**Figure 7:**
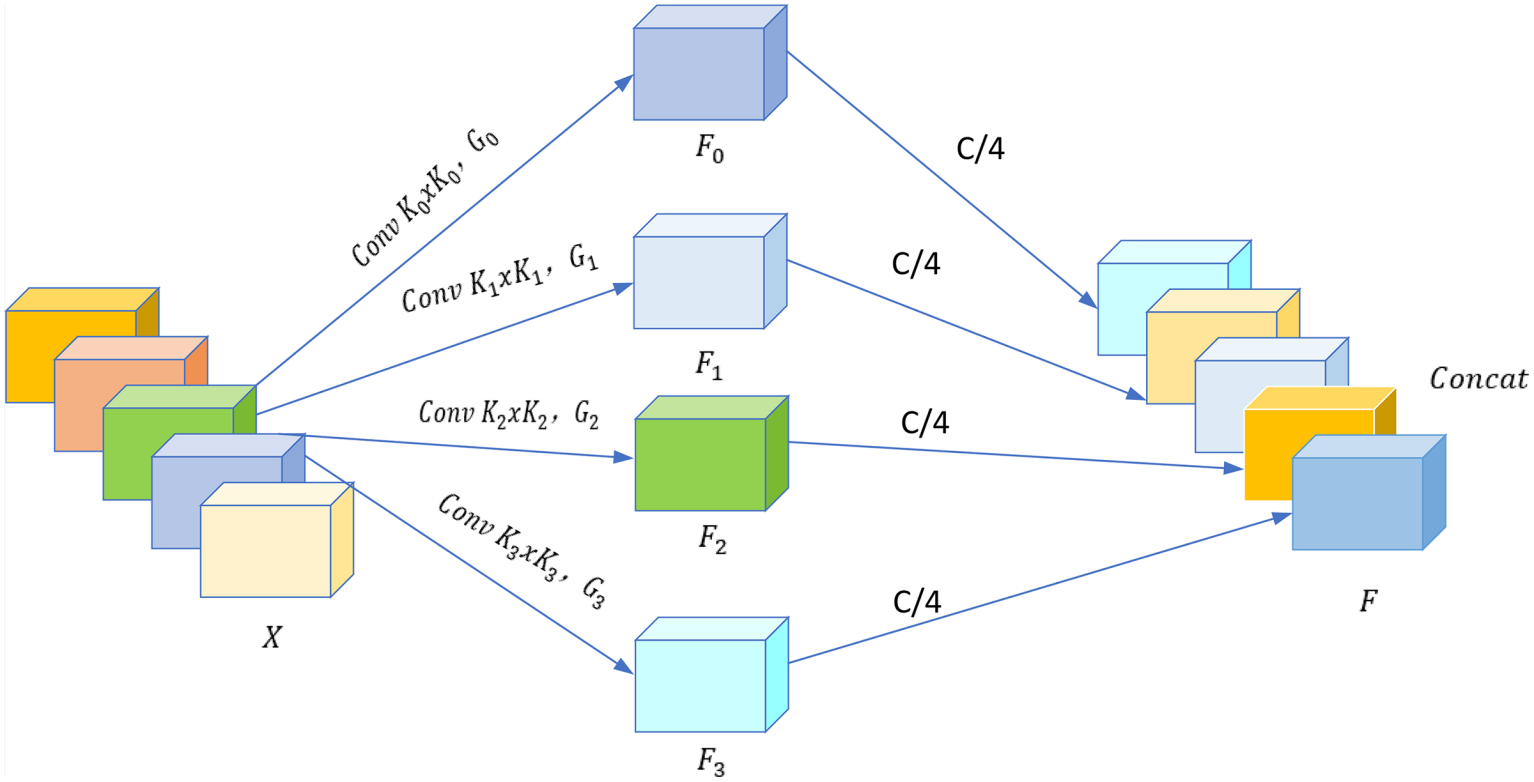
SPC module. Images © IAM Handwriting Database, non-commercial use only.

SPC is the key module of PSA to realize multi-scale feature extraction, the structure is shown in Fig. 7.

The multi-branch structure utilized by SPC is designed to extract spatial information from $X$, where $X$ represents the input feature map. Each individual branch within this structure operates with an input channel dimension of $C$. $X$ is sliced into $S$ parts, expressed as $\left[ {{X_0},{X_1}, \cdots ,{X_{S - 1}}} \right]$, and the number of channels in each part is ${C^\prime } = C/S$ (set $S$ = 4 in Fig. 7). After slicing, the feature map ${X_i} \in {{\mathbb R}^{{C^\prime } \times H \times W}}$ is obtained, where $i = 0,1, \cdots ,S - 1$. To extract this information effectively, multi-scale convolutions are utilized to produce feature maps with diverse spatial scales and depths. Every branch can learn the spatial information at various scales and creates localized interactions across channels. To better handle input tensors at different convolutional kernel scales, group convolution is applied to the kernels. The correlation between the convolutional kernel size at various scales and the group size can be expressed as

(5) $$G = {2^{\textstyle{{K - 1} \over 2}}}$$

where $K$ represents the convolution kernel size, $G\;$denotes the group size. The computation for feature extraction at various scales is determined as depicted in Eq. (6).

(6) $${F_i} = Conv({k_i} \times {k_i},{G_i})({X_i}),i = 0,1,2 \cdots S - 1$$

where the $i$- $th$ convolution kernel size is ${k_i} = 2 \times (i + 1) + 1$, and the size of $i$- $th$ group is ${G_i} = {2^{\textstyle{{{k_i} - 1} \over 2}}}$. ${F_i} \in {{\mathbb R}^{{C}^{\prime} \times H \times W}}$ signifies the feature maps at various scales. The finally obtained feature maps spanning multiple scales are connected by *Concat* way as shown in Eq. (7).

(7) $$F = Cat(\left[ {{F_0},{F_1}, \cdots ,{F_{S - 1}}} \right])$$

By deriving the attention weight vector from the feature map encompassing multiple scales, the attention weight vector can be represented as

(8) $${Z_i} = SEWeight({F_i}),\quad i = 0,1,2 \cdots S - 1$$

where ${Z_i} \in {{\mathbb R}^{{C^\prime } \times 1 \times 1}}$ represents the attention weight. The attention weight at various scales is obtained by the SEWeight module, while the PSA unit integrates contextual details from diverse scales, enhancing the pixel-level attention of the high-level feature maps. For the purpose of reinforcing the exchange of attention information, the cross-dimensional vectors are combined in a way that maintains the accuracy. The entire multiscale channel attention vectors are acquired in series as

(9) $$Z = {Z_0} \oplus {Z_1} \cdots \oplus {Z_{S - 1}}$$

where $\oplus$ represent the concatenation operator, ${Z_i}$ denotes the attention value ${F_i}$, and $Z$ is the vector of attention weights at various scales. Softmax is employed to readjust the weights of channel attention data, aiding in the creation of channel attention connections and improving information exchange with different scales, and the calculation process can be represented as

(10) $$at{t_i} = Softmax({Z_i}) = \displaystyle{{exp({Z_i})} \over {\sum\limits_{i = 0}^{S - 1} {exp({Z_i})} }}$$

The weights of multi-scale channels $at{t_i}$ are acquired through Softmax, which encapsulates attention weights of the channels and the positional details in space. This facilitates the realization of interaction between global and local channel attention. Following the channel attention is spliced and fused, and finally the complete channel attention vector can be acquired as

(11) $$att = at{t_0} \oplus at{t_1} \oplus \cdots \oplus at{t_{S - 1}}$$

in the attention interaction, $att$ represents the weights assigned to the multi-scale channels. Subsequently, the feature maps ${F_i}$ are multiplied by the channel attention weight $at{t_i}$, as shown in Eq. (12).

(12) $${Y_i} = {F_i} \odot at{t_i},i = 1,2,3, \cdots ,S - 1$$

where $\odot$ denotes the channel-wise multiplication, ${Y_i}$ signifies the feature map incorporating channel attention weight with different scales. The features obtained by splicing can be written as

(13) $$Out = Cat(\left[ {{Y_0},{Y_1}, \cdots ,{Y_{S - 1}}} \right])$$

The PSA module has the ability to incorporate spatial information from multiple scales and different channel attentions into each feature group’s blocks. Utilizing the PSA module enhances the understanding of information interaction between global and local channel attention during feature extraction, which in turn enables us to perform multi-scale feature extraction on handwritten font samples, learn features consistent with the handwriting style of handwritten fonts, and generate more realistic fonts.

## Results

### Implementation details

We perform experiments on the IAM (Marti & Bunke, 2002) dataset^1^ 1The dataset can be downloaded at https://fki.tic.heia-fr.ch/databases/iam-handwriting-database, which comprises 9,862 text lines and over 60,000 English words written by 500 different authors. We select handwritten images from 340 authors for training, and images from the remaining 160 authors for testing. Additionally, we used the CVL (Kleber et al., 2013) dataset^2^ 2The dataset can be downloaded at https://cvl.tuwien.ac.at/research/cvl-databases/an-off-line-database-for-writer-retrieval-writer-identification-and-word-spotting/, comprising 311 authors and 101,069 words, to conduct experiments and calculate the FID values. We used 284 authors for training and the remaining 27 authors to test the results. We used the Python and experimented in the Pytorch environment, using A100 GPU server to train and test our model. The images are adjusted to a constant height of 64 pixels while maintaining the aspect ratio of the original image. The number of layers for both the Transformer encoder and decoder attention is set to 3, with each layer having 8 attention heads. Training employs the Adam optimizer and sets the learning rate to 2 × 10^−4^, batch size set to 8. In this scenario, training halts after 9.8 k epochs.

### Evaluation metrics

The Fréchet inception distance (FID) (Heusel et al., 2017) score and the Geometry Score (GS) (Khrulkov & Oseledets, 2018) are used to evaluate the effectiveness of our model. FID is a metric used to calculate the distance between the features of the generated image and the features of the real image. A lower value of FID means that the features of the two are closer together, indicating that the generated font image is closer to the real font image. GS is a metric that calculates the topological similarity of the images. A lower GS value represents a higher quality of the image generated by the model. SSIM (Wang et al., 2004) and RMSE are metrics used to measure the preservation of pixel details in generated images. A higher SSIM indicates less distortion in the generated image. Additionally, the perceptual similarity of the generated image is quantified using LPIPS (Zhang et al., 2018), the lower LPIPS values indicate that the generated images are more realistic. The Handwriting Distance (HWD) (Pippi et al., 2023) metric operates in a specially trained network feature space, extracting handwriting style features from variable-length input images and comparing the subtle geometric features of handwritten fonts based on perceptual distance. A lower value of the HWD metric indicates a more realistic and authentic generated font image.

### Experiments

We evaluate our model using the test set from the IAM dataset, obtained FID and GS scores, and performed quantitative comparisons with the current more advanced HiGAN (Gan & Wang, 2021), ScrabbleGAN (Fogel et al., 2020), TS-GAN (Davis et al., 2020), HWT (Bhunia et al., 2021), CG-GAN (Kong et al., 2022), and VATr (Pippi, Cascianelli & Cucchiara, 2023) models in handwriting font generation research. For comparison, the results are presented in Table 1.

**Table 1:** Comparison of scores of different models for generating font image quality on the IAM test set. Best results are shown in bold.

Model	FID↓	GS↓
HiGAN (Gan & Wang, 2021)	24.90	3.19 × 10^−2^
ScrabbleGAN (Fogel et al., 2020)	20.72	2.56 × 10^−2^
TS-GAN (Davis et al., 2020)	20.65	4.88 × 10^−2^
HWT (Bhunia et al., 2021)	19.40	**1.01 × 10** ^**−2**^
CG-GAN (Kong et al., 2022)	19.03	–
VATr (Pippi et al., 2023)	17.79	1.68 × 10^−2^
PSA-HWT	**14.88**	1.46 × 10^−2^

According to Table 1, PSA-HWT obtained the best FID score, while GS score comes second with a slight difference from the optimal GS score. The FID score shows a decrease of 23.29% compared to the classical HWT model. The VATr model has FID score of 17.79 and a GS score of 1.68 × 10^−2^. The PSA-HWT model shows a decrease of 16.35% in FID score and 13.09% in GS score compared to the VATr model. Compared to CG-GAN, the FID value decreased by 21.81%. It demonstrates the effectiveness of pyramid squeeze attention in the model and how multi-scale feature extraction enables the generated handwritten font images to be closer to real images. As a result, the quality of generated fonts has been significantly improved.

Introducing pyramid squeeze attention into the model not only enables multi-scale feature extraction during feature extraction but also results in generated fonts that are more similar to real fonts. Table 1 provides a comparison of the font image quality scores. Additionally, this improves the generalization performance of the model significantly. We tested the FID values for the generated fonts under four settings: (1) Generate words from the training set using the style of authors from the training set (IV-S), (2) Generate words from the training set using the style of authors from the test set (IV-U), (3) Generate words using the style of authors from the training set for words that are not appearance in the training set (OOV-S), (4) Generate words using the style of authors from the test set for words that are not appearance in the training set (OOV-U). We compared the results with the GANwriting, TS-GAN, HWT, and CG-GAN models. From the data in Table 2, it can be seen that our model performed the best, particularly in the most challenging OOV-U setting.

**Table 2:** The comparative results of FID scores under four different settings. Best results are shown in bold.

Model	IV-S↓	IV-U↓	OOV-S↓	OOV-U↓
GANwriting (Kang et al., 2020)	120.07	124.30	125.87	130.68
TS-GAN (Davis et al., 2020)	118.56	128.75	127.11	136.67
HWT (Bhunia et al., 2021)	106.97	108.84	109.45	114.10
CG-GAN (Kong et al., 2022)	**102.18**	110.07	104.81	113.01
PSA-HWT	104.22	**105.86**	**104.78**	**107.07**

In the IV-S and IV-U settings, compared to the HWT model, the FID values decreased by 2.57% and 2.74%, respectively. It can be observed that in the most challenging OOV-U setting, our model achieved better FID scores compared to other models on the IAM dataset. Specifically, the FID score is 5.25% lower than the best-performing CG-GAN. This demonstrates that our model has better performance and generalization.

**Figure 8:**
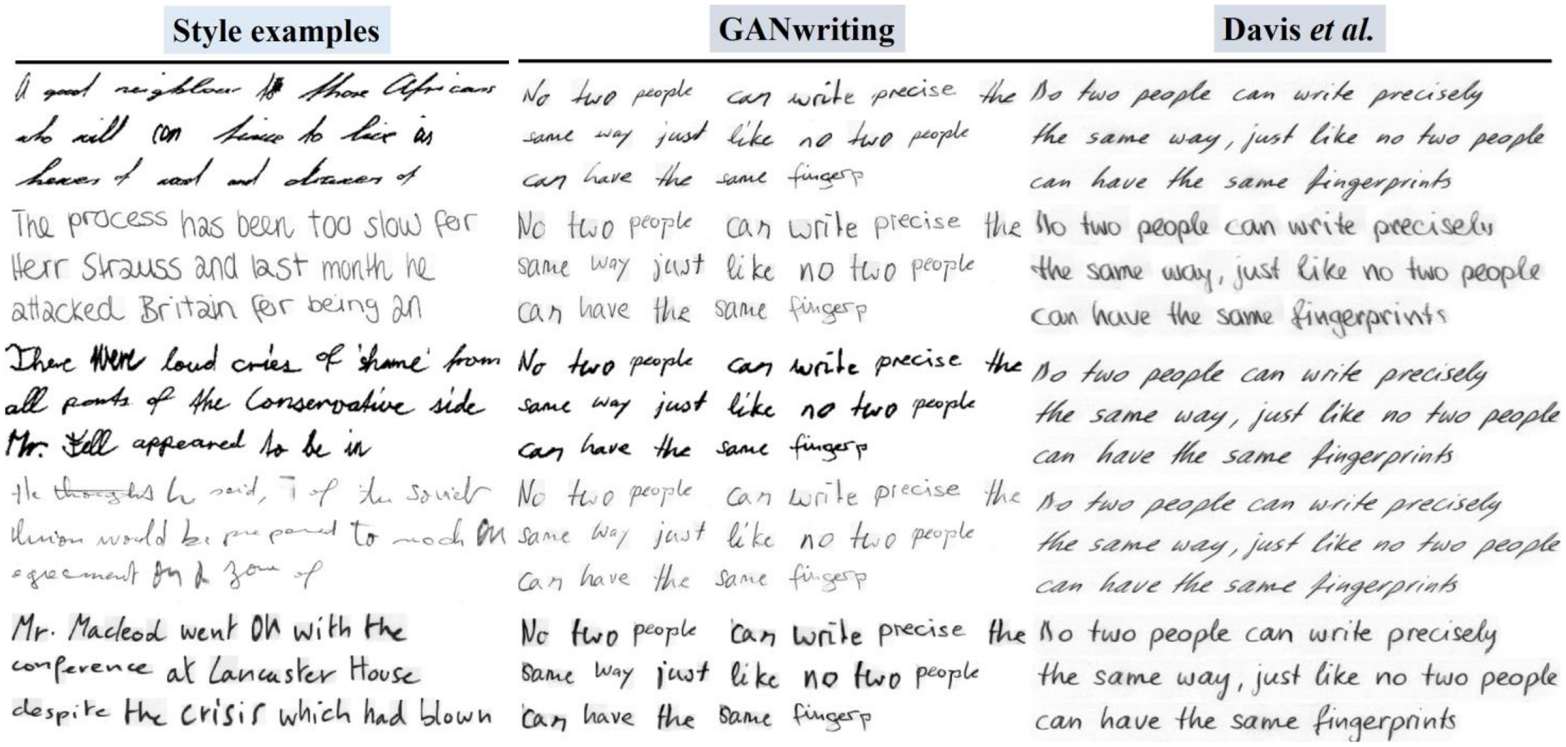
GANwriting and Davis. Images © IAM Handwriting Database, non-commercial use only.

**Figure 9:**
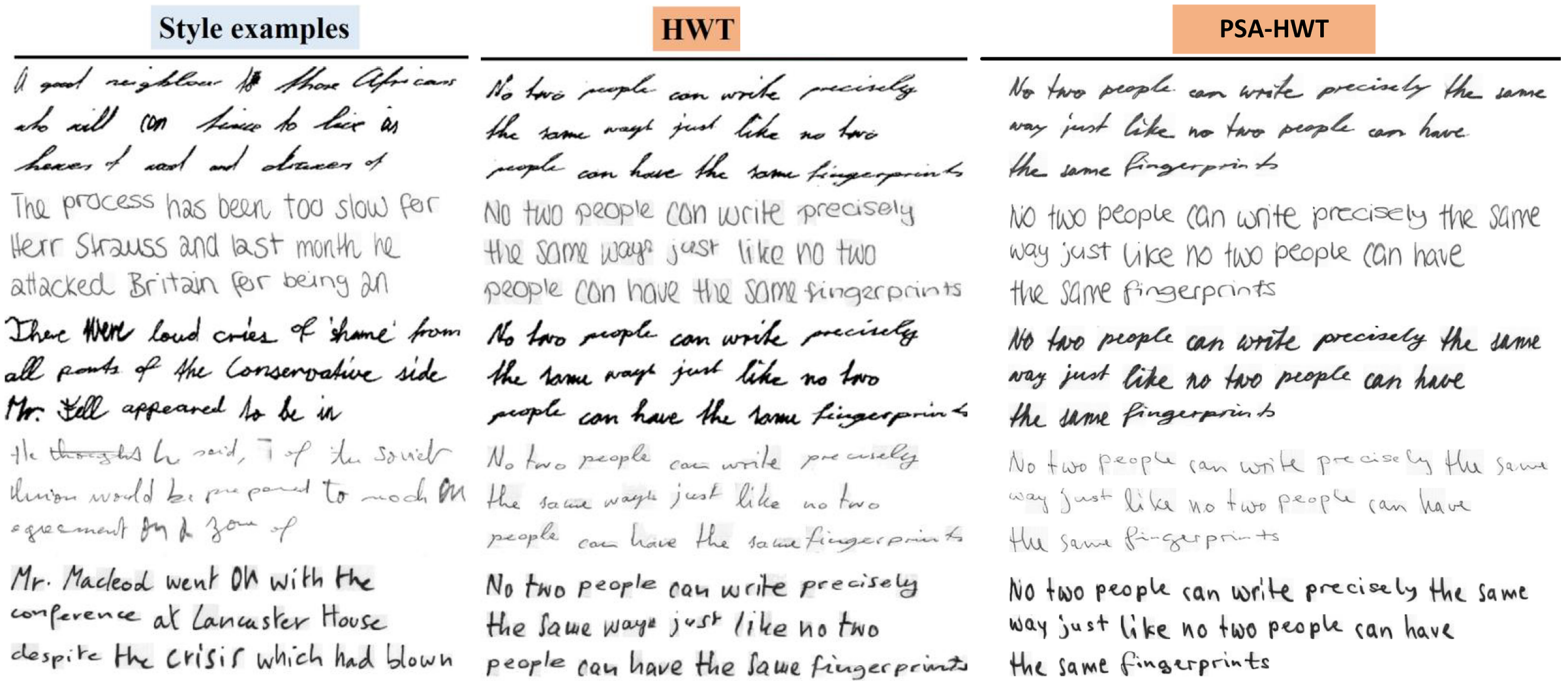
HWT and PSA-HWT. Images © IAM Handwriting Database, \non-commercial use only.

To demonstrate the performance advantage of our model, we conducted a qualitative comparison with HWT, GANwriting, and Davis. We use the same text content to evaluate the quality of the generated fonts for all four methods. The initial column displays various writers’ examples of stylistic choices (see Figs. 8 and 9). (Note that all images in Figs. 8 and 9 except our model are from Bhunia et al., 2021)

The first column represents examples of different authors’ styles. Although the HWT model can mimic the global and local style patterns of fonts, it cannot capture more fine-grained font features such as the curvature and thickness of strokes. Due to being restricted to a fixed length of query words, GANwriting cannot complete the provided text content, and it has difficulty adhering to the left-tilted top and character style of the examples. The model by Davis et al. can capture the global style but has difficulty imitating the stylistic details of specific characters. This generated font follows the left tilt of a style example but struggles to capture character-level styles and cursive patterns (*e.g*., the word “the”). Our model not only better simulates global and local style patterns but also captures the fine-grained font features of the examples, generating realistic handwritten text images.

To further validate that introducing pyramid squeeze attention into the HWT model can sufficiently extract multi-scale features. We evaluate the model using evaluation metrics for handwriting font generation quality, including HWD, as well as commonly used metrics such as SSIM, RMSE, and LPIPS, with results compared to the HWT model as shown in Table 3.

**Table 3:** Comparison of model scores. Best results are shown in bold.

Model	SSIM↑	RMSE↓	LPIPS↓	HWD↓
HWT	0.3441	**5.5708**	0.2776	0.5521
PSA-HWT	**0.3528**	5.5817	**0.2678**	**0.4118**

As shown in Table 3, compared to the HWT model, the SSIM value increased by 2.46%, the LPIPS value decreased by 3.53%, and the HWD value decreased by 25.41% in terms of font style comparison. This demonstrates that the PSA-HWT model outperforms the HWT model in generating detailed font pixels, aligning with human visual perception, and capturing subtle geometric features of fonts.

To assess the generalization capabilities of the PSA-HWT model, we employed the IAM dataset as the training set and the CVL dataset as the test set. The generated fonts were then evaluated quantitatively using the HWT model, as depicted in Table 4. KID (Bińkowski et al., 2018) is a GAN generation metric used to evaluate the quality of the generated image. A lower KID value indicates better convergence and higher image quality. Comparing our model to the HWT model, our model demonstrates better performance in terms of KID value and HWD value, with a 30.60% decrease in KID score and a 10.69% decrease in HWD score. This highlights the effectiveness of pyramid squeeze attention in our model. Additionally, our model shows a 21.46% decrease in FID value and a 49.27% decrease in GS value compared to the HWT model. This suggests that the handwritten fonts generated by our model closely resemble real handwritten fonts in terms of style and realism. Furthermore, our model demonstrates a significant improvement in generalizability.

**Table 4:** Quantitative comparison of generalization experiments. Best results are shown in bold.

Model	FID↓	KID↓	HWD↓	GS↓
HWT	46.08	2.81 × 10^−2^	0.5527	0.69 × 10^−2^
PSA-HWT	**36.19**	**1.95 × 10** ^**−2**^	**0.4936**	**0.35 × 10** ^**−2**^

We also conducted experiments by training our model using the CVL dataset and calculated the FID values for the test results. The comparative results are shown in Table 5. The FID and GS values decreased by 10.04% and 38.46%, respectively. The HWD and KID scores decreased by 6.35% and 13.88%, respectively.

**Table 5:** Comparison of model scores. Best results are shown in bold.

Model	FID↓	KID↓	HWD↓	GS↓
HWT	18.72	0.36 × 10^−2^	0.3477	0.91 × 10^−2^
PSA-HWT	**16.84**	**0.31 × 10** ^**−2**^	**0.3256**	**0.56 × 10** ^**−2**^

### Analysis

**Figure 10:**
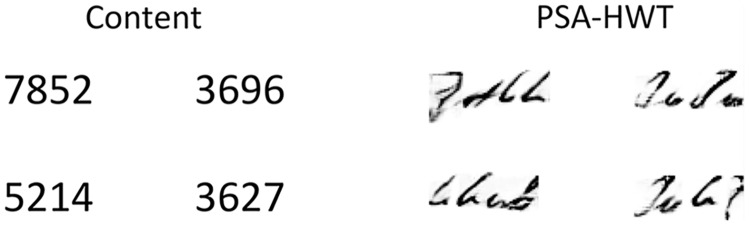
Failure cases. Images © IAM Handwriting Database, non-commercial use only.

Although the model performs well in generating handwritten fonts, it does not perform well in generating digits (see Fig. 10). This may be due to the following reasons: the content features of the font are extracted from the one-hot representation of the query. Since one-hot encoding can only handle known categories, it cannot effectively encode unknown categories. Therefore, when generating unseen categories in the test set, one-hot encoding encounters issues, resulting in generated content that does not meet expectations.

## Discussion

Although the HWT model has achieved good performance in generating handwritten fonts using a combination of CNN and Transformer, it still faces limitations in extracting features at different scales such as stroke thickness and curvature. This results in a noticeable gap between the generated fonts and real handwritten fonts. In contrast, our model addresses this issue by introducing pyramid squeeze attention, enabling multi-scale feature extraction and generating more realistic handwritten fonts. While our model demonstrates excellent performance, it also has some shortcomings. While the attention mechanism can enhance the model’s ability to focus on crucial features like stroke position, shape, and curvature, it does not effectively address stroke coherence. Future research will research this aspect to enhance the quality of handwritten fonts. On one hand, research on handwritten font generation will have beneficial impacts on future research and potential applications. For example, it can be used for personalized and customized font design. Future studies can explore how to generate customized fonts based on users’ needs and preferences to meet the demands of different user groups, such as designers, brands, and individual users. Moreover, it can be applied in educational settings and other fields. Future research can explore how to use generated handwritten font samples to develop educational tools and assistive technologies to help students improve their handwriting and reading comprehension skills. On the other hand, research on handwritten font generation must ensure that the generated fonts are not used for purposes of discrimination, defamation, or infringement of others’ rights. We should consider how our results impact society and how to ensure their fair and reasonable use.

## Conclusion

To address the limitations of the current model in effectively extracting fine-grained features such as stroke thickness and curvature of handwritten fonts during training, resulting in the generation of fonts that are inconsistent with the style of real handwritten fonts and not realistic enough, we propose a handwritten font generation approach called PSA-HWT. The model is based on pyramid squeeze attention and aims to extract spatial information from the input feature map for multi-scale feature extraction. By learning fine-grained features, including stroke thickness and curvature, PSA-HWT generates more realistic handwritten fonts. Numerous experiments have consistently shown that the pyramid squeeze attention enables the encoder to efficiently extract finely detailed features at various scales, resulting in handwritten fonts that are both realistic and consistent with real handwriting styles. However, further enhancements to the realism of these fonts remain an area for future research.

## Supplemental Information

10.7717/peerj-cs.2261/supp-1 Supplemental Information 1 Supplemental Material.

10.7717/peerj-cs.2261/supp-2 Supplemental Information 2 Code.

## Additional Information and Declarations

### Data Availability

The following information was supplied regarding data availability: The raw data and code files are available in the Supplemental Files. Raw data is also available at University of Bern and the TU Wien: https://fki.tic.heia-fr.ch/databases/iam-handwriting-database.
https://cvl.tuwien.ac.at/research/cvl-databases/an-off-line-database-for-writer-retrieval-writer-identification-and-word-spotting/
