# Supplementary material for: PSA-HWT: handwritten font generation based on pyramid squeeze attention
Source: PeerJ Comput Sci. 2024 Aug 23;10:e2261. doi: 10.7717/peerj-cs.2261 (PMC11639151; doi:10.7717/peerj-cs.2261)
Supplement: Supplemental Information 1 [file peerj-cs-10-2261-s001.docx]

PSA-HWT: Handwritten Font Generation Based on Pyramid Squeeze Attention

**Supplementary Material**

In this supplementary material, we present more qualitative results on IAM dataset. Additionally, the literature is presented in tabular form, with the results shown in Table 1.

Table 1: Relevant literature on handwritten font generation

| Method | Article (Author) | Year |
| --- | --- | --- |
| stroke-based online | Graves et al. | 2013 |
|  | Kotani et al. | 2020 |
| image-based offline | Alonso et al. | 2019 |
|  | Chang et al. | 2018 |
|  | Davis et al. | 2020 |
|  | Fogel et al. | 2020 |
|  | Haines et al. | 2016 |
|  | Kang et al. | 2020 |
|  | Bhunia et al. | 2021 |
|  | Kong et al. | 2022 |
|  | Pippi et al. | 2023 |

Figure 1: Additional qualitative comparisons of our proposed PSA-HWT with HWT, when generating the same text ‘The self-confidence is the fundamental factor for our glorious personal success’.

Figure 2: Additional qualitative comparisons of our proposed PSA-HWT with HWT, when generating the same text ‘It is self-confidence that helps me create enormous values to the general public and fulfill the great personal ambition’.

Figure 3: Additional qualitative comparisons of our proposed PSA-HWT with HWT, when generating the same text ‘The absence of self-confidence will definitely lead to one's lower achievement and inadequate personal growth’.

Figure 4: Handwritten text image generation of arbitrarily long words. We generate the 26-letter word‘radioimmunoelectrophoresis’and compare the results with HWT.

Figure 5: Handwritten text image generation of arbitrarily long words. We generate the 30-letter word‘pseudopseudohypoparathyroidism’ and compare the results with HWT.
